# Degradation of Lipid Droplets in Plants and Algae—Right Time, Many Paths, One Goal

**DOI:** 10.3389/fpls.2020.579019

**Published:** 2020-09-09

**Authors:** Krzysztof Zienkiewicz, Agnieszka Zienkiewicz

**Affiliations:** Centre for Modern Interdisciplinary Technologies, Nicolaus Copernicus University in Toruń, Toruń, Poland

**Keywords:** lipid droplets (LDs), triacylglycerols (TAGs), lipid droplet degradation, lipolysis, lipase, autophagy, lipophagy

## Abstract

In eukaryotic cells, lipids in the form of triacylglycerols (TAGs) are the major reservoir of cellular carbon and energy. These TAGs are packed into specialized organelles called lipid droplets (LDs). They can be found in most, if not all, types of cells, from bacteria to human. Recent data suggest that rather than being simple storage organelles, LDs are very dynamic structures at the center of cellular metabolism. This is also true in plants and algae, where LDs have been implicated in many processes including energy supply; membrane structure, function, trafficking; and signal transduction. Plant and algal LDs also play a vital role in human life, providing multiple sources of food and fuel. Thus, a lot of attention has been paid to metabolism and function of these organelles in recent years. This review summarizes the most recent advances on LDs degradation as a key process for TAGs release. While the initial knowledge on this process came from studies in oilseeds, the findings of the last decade revealed high complexity and specific mechanisms of LDs degradation in plants and algae. This includes identification of numerous novel proteins associated with LDs as well as a prominent role for autophagy in this process. This review outlines, systemizes, and discusses the most current data on LDs catabolism in plants and algae.

## Introduction

**Figure 1:**
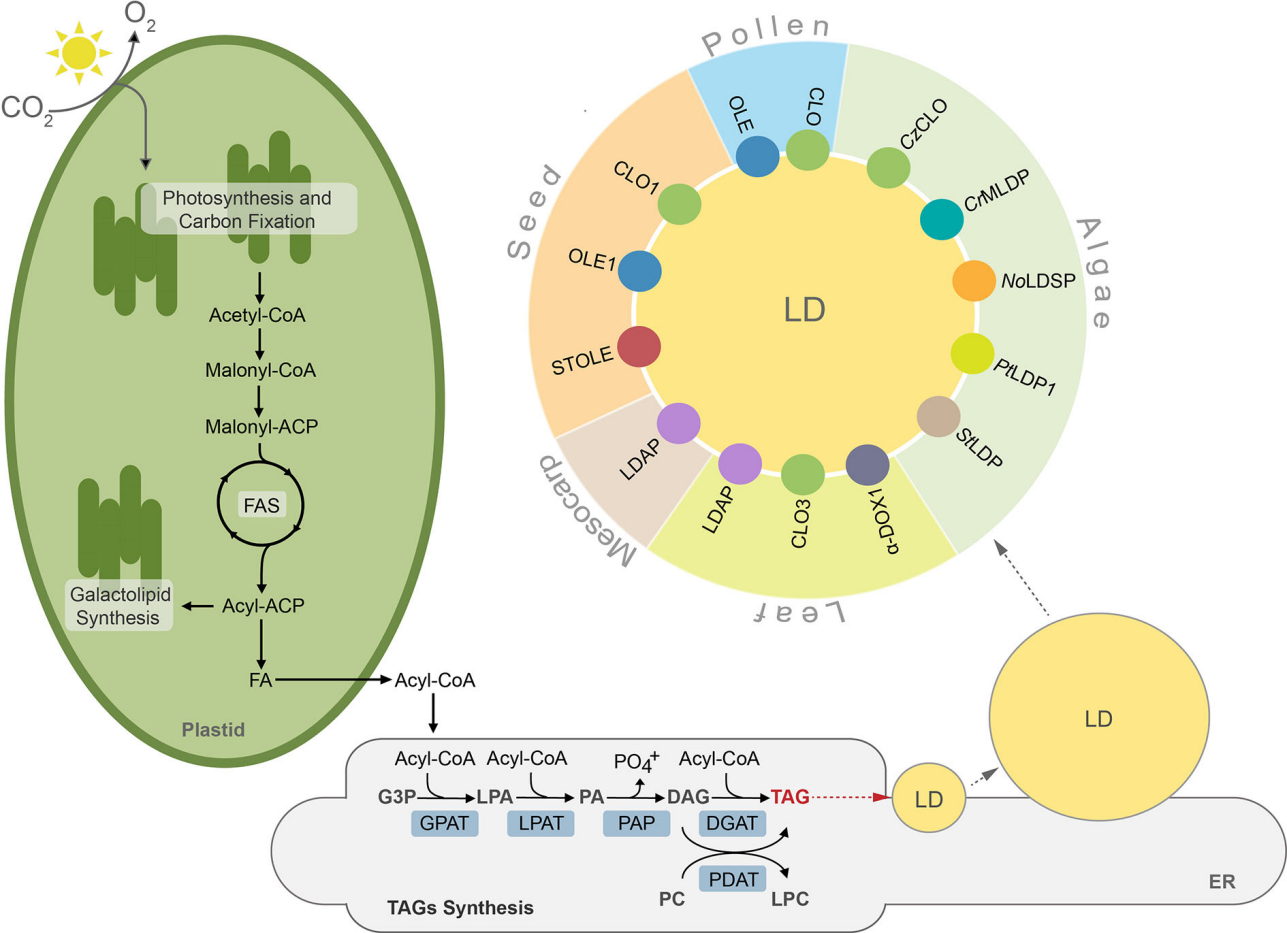
TAGs synthesis, lipid droplet formation and their protein equipment in plant and algal cells. ACP, acyl carrier protein; CoA, coenzyme A; CLO, caleosin; *Cr*MLDP *Chlamydomonas reinhardtii* major lipid droplet protein; *Cz*CLO, *Chromochloris zofingiensis* caleosin; DAG, diacylglycerol; DGAT, acylCoA:diacylglycerol acyltransferase, α-DOX1, dioxygenase 1; FA, fatty acid; FAS, fatty acid synthase, G3P, glycerol-3-phosphate; GPAT, acyl-CoA:glycerol-3-phosphate-acyltransferase; LD, lipid droplet; LDAP, lipid droplet associated protein; LPA, lysophosphatidic acid; LPAT, acyl-CoA:lysophosphatidic acid acyltransferase; LPC, lysophosphatidylcholine, *No*LDSP, *Nannochloropsis oceanica* lipid droplet surface protein; OLE, oleosin; PA, phosphatidic acid; PAP, phosphatidic acid phosphatase, PDAT, phospholipid:diacylglycerol acyltransferase; PC, phosphatidylcholine; *Pt*LDP1, *Phaeodactylum tricornutum* lipid droplet protein 1; StLDP, Stramenopile-type lipid droplet protein; STOLE, steroleosin; TAG, triacylglycerol.

Lipids in plants and algae can be generally divided into two major groups, storage lipids and membrane lipids. The former serve as energy and carbon reservoirs, and the latter are building blocks for photosynthetic and non-photosynthetic membranes (Li-Beisson et al., 2013). Additionally, land plants deposit a lipidic layer composed of waxes and cutin on their surface, which serves as an impermeable barrier protecting them from excessive water loss, pathogens, and toxins (Nawrath et al., 2013; Serrano et al., 2014). The storage lipids are represented mainly by triacylglycerols (TAGs) and to a much lesser degree by sterol esters, whereas lipid composition of membranes is more complex and differs between cell compartments, cell types, and organisms (Bouvier-Navé et al., 2010; Horn et al., 2011; Goold et al., 2015; Shimada et al., 2019). Broadly, galactolipids are much more abundant in plastidial membranes, whereas non-plastidial membranes are composed mostly of phospholipids (Li-Beisson et al., 2013). Synthesis of lipids in plant and algal cells starts in plastids, where fatty acids (FAs) are generated. These molecules are further used as substrates in the key cellular pathways of membrane lipid and TAGs synthesis. Before entering complex metabolic pathways FAs often undergo modifications, like desaturation or elongation. Regardless of their modification level, FAs can reside in the chloroplast, where they function as substrates for synthesis of plastidial membrane lipids, like monogalactosyldiacylglycerol (MGDG) or digalactosyldiacylglycerol (DGDG) (Wang and Benning, 2012; Lavell and Benning, 2019). When exported to the cytosol, free FAs are first exported by FATTY ACID EXPORT1 (FAX1) across the chloroplast inner envelope and then undergo vectorial acylation by the chloroplast outer envelope-localized long-chain acyl-CoA synthetase 9 (LACS9) (Schnurr et al., 2002; Li et al., 2015; Li et al., 2016; Li et al., 2019). The resulting conjugates of FAs with coenzyme A (CoA)—acyl-CoAs are transported into endoplasmic reticulum (ER) and incorporated into the cellular pool of carbon and energy in the form of TAGs (Li et al., 2016). The major pathway of TAGs synthesis is the glycerol-3-phosphate (G-3-P) (or Kennedy) pathway (**Figure 1**) (Ohlrogge and Browse, 1995; Chapman and Ohlrogge, 2012). In this pathway, G-3-P is successively esterified with acyl chains from acyl-CoAs. These reactions occur in a precise manner and are catalyzed by specific acyltransferases located in the endoplasmic reticulum (ER) membrane. In the first reaction, catalyzed by glycerol-3-phosphate acyltransferase (GPAT), G-3-P is linked to acyl-CoA and lysphosphatidic acid (LPA) is generated. LPA is then converted into phosphatidic acid (PA) by esterification with another acyl-CoA. This reaction is catalyzed by lysophosphatidate acyltransferase (LPAT). PA can serve as a substrate for synthesis of phospholipids or TAGs. In the latter case, dephosphorylation of PA by phosphatidic acid phosphatase (PAP) leads to formation of diacylglycerol (DAG) (Li-Beisson et al., 2013). The synthesized DAG is further converted into TAG by acyl-CoA:diacylglycerol acyltransferases (DGATs), and this reaction is considered a committed step of TAG formation (Zhang et al., 2009; Turchetto-Zolet et al., 2011; Sanjaya et al., 2013; Zienkiewicz et al., 2016; Zienkiewicz et al., 2017; Zienkiewicz et al., 2018). In addition to DGAT-mediated pathway DAG can also be acylated into TAG by the phospholipid:diacylglycerol acyltransferase (PDAT) (**Figure 1**). This enzyme has been shown to synthesize TAG *via* acyl-CoA-independent transacylation of DAG using phosphatidylcholine (PC) as acyl donor (**Figure 1**) (Dahlqvist et al., 2000; Zhang et al., 2009; Yoon et al., 2012).

TAGs synthesized in the ER membrane are continuously deposited between leaflets of the membrane bilayer, finally forming spherical organelles referred to in the literature by many terms, such as lipid droplets (thereafter LDs), oil bodies (OBs), oleosomes, or spherosomes (**Figure 1**). A prominent role for LDs formation at ER membrane in plants has been shown for SEIPIN proteins (Cai et al., 2015; Taurino et al., 2018; Greer et al., 2020). Indeed, loss of function *Arabidopsis thaliana* SEIPIN isoforms as well as their overexpression results in altered size of LDs, however, the exact role of these proteins in LDs formation remains to be clarified (Cai et al., 2015; Taurino et al., 2018). Recent studies showed also that mutation in the gene encoding *At*VAP27-1 (Vesicle-Associated Membrane Protein (VAMP)–Associated Proteins (VAPs)) resulted in formation of aberrant and enlarged LDs. Moreover, a direct interaction between Arabidopsis SEIPIN isoforms (2 and 3) and *At*VAP27-1 indicates that these two proteins most probably cooperate during formation of LDs (Greer et al., 2020). A putative SEIPIN ortholog has also been identified in diatom *Phaeodactylum tricornutum.* Overexpression of this SEIPIN in *P. tricornutum* resulted in biogenesis of larger LDs, suggesting similarities in the functional nature of SEIPINs between plants and algae (Lu et al., 2017).

After their complete formation, LDs separate from the ER membrane and localize to the cytosol (Chapman and Ohlrogge, 2012; Murphy, 2012; Olzmann and Carvalho, 2019). LDs have been identified in cells of diverse organisms, including bacteria (Zhang et al., 2017), yeast (Grillitsch et al., 2011), algae (Zienkiewicz et al., 2020), plants (Tzen et al., 1993), nematodes (O’Rourke et al., 2009), and mammals (Birsoy et al., 2013). This indicates their highly conserved role in cellular lipid metabolism. Until recently LDs were considered as a simple TAG storage compartment, however, intense studies in the last decade revealed that LDs represent highly dynamic structures, involved in a plethora of diverse cellular processes, like regulation of energy homeostasis, remodeling of membranes, and signaling (Chitraju et al., 2017; Welte and Gould, 2017; Yang and Benning, 2018; Fernandez-Santos et al., 2020). According to the common structural model, LDs are composed of a hydrophobic core filled mostly with TAGs surrounded by a phospholipid monolayer, and are decorated with a set of specific proteins (**Figure 1**). These proteins are considered essential for biogenesis, stabilization, and mobilization of LDs, and are cell-, tissue-, and organism-specific (**Figure 1**, **Table S1**) (Tzen et al., 1993; Huang, 2018).

## Lipid Droplets—Multiple Variants Of The Same Organelle?

In plants, structural proteins of LDs were first and best characterized in seeds (Tzen et al., 1990; Tzen et al., 1993; Jolivet et al., 2004; Huang, 2018; Du et al., 2019). The set of major LD structural proteins found in seeds includes oleosin, caleosin, and steroleosin, however their individual isoforms are also present in non-seed tissues (**Table S1**) (Chapman et al., 2012; Huang, 2018). Oleosins are the most abundant integral membrane proteins of oilseed LDs. They are anchored in the phospholipid monolayer by a hydrophobic α-helical hairpin domain with a proline knot, and their C- and N- termini face the cytosol (Abell et al., 1997; Napier et al., 2001; Alexander et al., 2002). As *Arabidopsis thaliana* mutant of one of the seed-specific oleosins *ole1* accumulates larger LDs when compared to wild type plants, it has been proposed that oleosins control the structure and size of LDs by preventing their uncontrolled fusion (Siloto et al., 2006; Shimada et al., 2008). Caleosins are much less abundant compared to oleosins in the LDs fraction from oilseeds. Their name derives from the ability to bind calcium ions (by a single EF-hand binding motif) and structural similarity to oleosins (Chen et al., 1999). Phylogenetic studies so far suggest that caleosin proteins were likely the ancestors of oleosins, as the putative genes encoding for caleosin-, but not oleosin-like proteins, are present in algae, non-vascular plants, and fungi (Jiang and Tzen, 2010; Rahman et al., 2018a; Rahman et al., 2018b). The ability to bind calcium ions, together with the presence of several phosphorylation sites and possession of peroxygenase activity suggest that caleosins mediate signaling between diverse developmental and stress signals and LDs (Hanano et al., 2006; Purkrtova et al., 2008). Unlike caleosins and oleosins, steroleosins possess only two structural motifs: an N-terminal hydrophobic region responsible for association with LD membranes through conserved proline residues, and a C-terminal domain with hydroxysteroid dehydrogenase (HSD) activity (d’Andrea et al., 2007). Steroleosins are thought to play a role in brassinosteroid-mediated cellular signaling during plant growth and development (Lin et al., 2002). Recent advances in LDs proteomics identified many new LD-associated proteins in seeds and seedlings, however their functions remain to be deciphered (Zhi et al., 2017; Kretzschmar et al., 2020).

After the seed, pollen grains are one of the most active sites in TAGs biosynthesis (Piffanelli et al., 1998; Zienkiewicz et al., 2014a). Mature pollen grains of many plants, especially oleaginous species, accumulate a high number of LDs in the cytoplasm of the vegetative cell (Zienkiewicz et al., 2014a). Similar to seeds, pollen LDs are coated with oleosin and caleosin, but no pollen steroleosin has yet been identified (Kim et al., 2002; Jiang et al., 2008; Zienkiewicz et al., 2010; Zienkiewicz K. et al., 2011). Interestingly, oleosin-like and caleosin proteins are also present in the pollen coat (Mayfield et al., 2001; Rejon et al., 2016), anther loculus, as well as in tapetal cells (Zienkiewicz K. et al., 2011; Levesque-Lemay et al., 2016). The latter tissue is directly involved in pollen development and is extremely rich in lipidic structures known as tapetosomes. They are released from degrading tapetum during anther development and targeted to the surface of developing pollen grains, eventually forming the pollen coat (Parish and Li, 2010; Levesque-Lemay et al., 2016). Interestingly, each tapetosome consists of multiple LDs clustered together and coated by oleosins (Hsieh and Huang, 2005) and/or caleosin (Zienkiewicz K. et al., 2011). This could explain the presence of both these proteins in the pollen coat (**Table S1**) and suggests that mature pollen grains are equipped with LD-associated proteins of both gametophytic and sporophytic origin.

Depending on developmental stage and/or physiological state of the plant, LDs can also be present in non-seed organs such as fruits, leaves, stems, and roots (Lersten et al., 2006; Shimada et al., 2015; Pyc et al., 2017a; Huang, 2018). Leaf LDs seem to be equipped with a different set of integral proteins than seeds (**Table S1**) (Pyc et al., 2017a; Fernandez-Santos et al., 2020). A leaf-specific isoform of caleosin, CLO3, has been shown to decorate leaf LDs and to be directly involved in triggering the oxylipin-mediated response to pathogen attack in *A. thaliana* (Hanano et al., 2015; Shimada et al., 2015). Infection of Arabidopsis by the pathogenic fungus *Colletotrichum higginisianum* leads to localization of α-dioxygenase 1 (α-DOX1) on the surface of leaf LDs and the formation of 2-hydroperoxy-octadecatrienoic acid (2-HPOT) from α-linolenic acid released from TAGs stored in LDs. 2-HPOT is then converted by CLO3 into 2-hydroxy-octadecatrienoic acid (2-HOT), which possesses anti-fungal activity (Shimada et al., 2014). Additionally, accumulation of phytoalexin deficient 3 (PAD3) protein involved in camalexin synthesis was observed on the surface of leaf LDs after *Pseudomonas syringae* pv *tomato* (*Pst*) DC3000 *avrRpm1* infection, supporting the role of leaf LDs in the plant defense response (Fernandez-Santos et al., 2020). Besides caleosin, three isoforms of Lipid Droplet Associated Proteins (LDAPs) have been identified in the leaves of Arabidopsis. LDAPs have been shown to be essential for the maintenance and regulation of LD metabolism and to play nonredundant functions in the stress response and post-germinative growth (Gidda et al., 2016). In addition to the above mentioned LD-associated proteins, a few proteins with diverse functions were also identified in the proteome of leaf LDs, including LDAP-interacting protein (LDIP) (Pyc et al., 2017b), glycerol-3-phosphate-acyltransferase 4 (GPAT4) and 8 (GPAT8) (Fernandez-Santos et al., 2020), strictosidine synthase (STR), 2-oxoglutarate (2OG) and farnesylcysteine lyase (FCLY) (Brocard et al., 2017). These observations suggest the multifunctional nature of LDs and support the direct involvement of non-seed LDs in stress-response and defense-related pathways in plants. Among non-seed tissues, few but extremely large LDs (over 10 µm of diameter) have also been found in mesocarp cells of some oleaginous fruits, like olive and avocado (Horn et al., 2013; Bartolini et al., 2014). Similar to seed LDs, mesocarp LDs are filled with TAGs, but they mostly lack oleosin and are equipped with LDAP protein instead (Horn et al., 2013; Zhi et al., 2017).

Under favorable growth conditions, algae usually accumulate small amounts of TAGs, whereas upon stresses such as nutrient limitation (e.g. nitrogen (N) deprivation), elevated temperatures, or high light intensities they synthesize massive amounts (Goold et al., 2015; Li-Beisson et al., 2015; Zienkiewicz et al., 2016). Algal LDs are equipped with specific LD-associated proteins, different from their plant counterparts (Moellering and Benning, 2010; Vieler et al., 2012a; Zienkiewicz et al., 2016; Leyland et al., 2020). The major LD protein MLDP was first identified in green algae, *Chlamydomonas reinhardtii* (Moellering and Benning, 2010; Nguyen et al., 2011) and then in *Dunaiella salina* (Davidi et al., 2012), *Scenedesmus quadricauda* (Javee et al., 2016), *Chromochloris zofingiensis* (Wang et al., 2019), and *Lobosphaera incisa* (Siegler et al., 2017). MLDPs are different from oleosin in lacking the central hydrophobic region, and appear to be specific to the lineage of green algae. Similar to structural proteins covering LDs in land plants, *Cr*MLDP also seems to be involved in the control of LD size and stabilization (Tsai et al., 2015). In turn, LDs of the oleaginous alga *Nannochloropsis oceanica* are decorated with Lipid Droplet Surface Protein (LDSP), which has been shown to localize on the LD surface during N deprivation (Vieler et al., 2012a; Vieler et al., 2012b; Zienkiewicz et al., 2020). *No*LDSP protein, similar to plant oleosin, possesses a proline-rich hydrophobic domain in its central region, however the proline knot motif found in oleosin is absent in *No*LDSP (Vieler et al., 2012a). Major LD structural proteins were also identified in the diatom *P. tricornutum*, including LD-associated protein (*Pt*LDP1) (Wang et al., 2017), a homolog of oleosome-associated-protein 1 (DOAP1) from *Fistulifera solaris* (Maeda et al., 2014), and Stramenopile-type lipid droplet protein (StLDP) (Yoneda et al., 2016). Overexpression of both these proteins in *P. tricornutum* was correlated with increased TAGs content and enlarged LDs during N deprivation (Yoneda et al., 2016; Wang et al., 2017), suggesting their analogous role to plant oleosin. Recently, three caleosin-related proteins were identified by proteomic analyses of LDs from *C. zofingiensis*, and co-localization for two of them on LDs was confirmed in *Saccharomyces cerevisiae* (Wang et al., 2019). Besides the major structural proteins detected in algal LDs, multiple proteomic studies have been performed to identify new LD proteins in diverse algal strains (Siegler et al., 2017; Lupette et al., 2019; Wang et al., 2019), however their specific localization and potential role in LD homeostasis have yet to be investigated.

Regardless of LD protein and TAG composition, their mobilization is crucial for providing energy and carbon during periods of active metabolism. This process is highly coordinated with development (e.g. seed germination) as well as responses to specific environmental conditions (e.g. nutrient deprivation). The release of TAGs from LDs requires the coordinated action of molecular machineries governing protein and lipid breakdown. Below we characterize the current state of knowledge regarding these pathways and interactions.

## LDs Lipolysis—Tag Lipases and Co-Workers

TAG lipases play one of the most essential roles in LDs degradation in plants and algae. These enzymes hydrolyze TAGs stored in LDs leading to the release of FAs, DAGs, MAGs, and glycerol (Graham, 2008; Kelly and Feussner, 2016). Glycerol is subsequently phosphorylated, oxygenated, and enters into glycolysis, whereas FAs undergo *β*-oxidation into acetyl-CoAs (Eastmond and Graham, 2001). In plants (Graham, 2008; Pracharoenwattana et al., 2010; Rinaldi et al., 2016) and green algae (Kong et al., 2018a; Kong et al., 2018b) the latter process takes place in peroxisomes (glyoxysomes), meanwhile in diatoms both mitochondrial and peroxisomal FAs *β*-oxidation is tough to occur (Chauton et al., 2013; Jallet et al., 2020).

**Figure 2:**
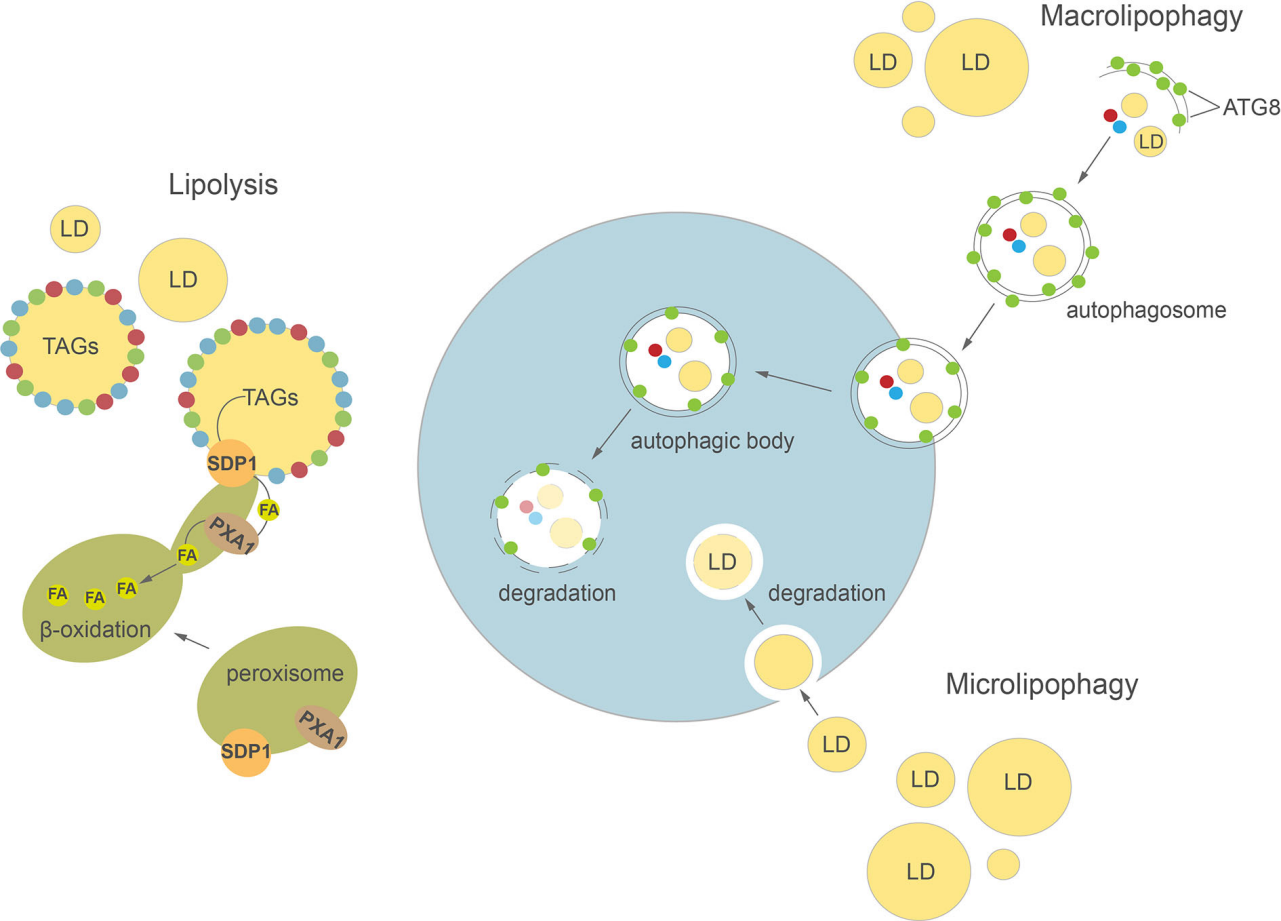
Cellular pathways of lipid droplets degradation in plants and algae. Detailed description in the text. ATG8, AUTOPHAGY-RELATED PROTEIN 8; FA, fatty acid; LD, lipid droplet; SDP1, SUGAR DEPENDENT 1; TAGs, triacylglycerols.

So far, the TAG lipases directly involved in LD mobilization are best characterized in plants, mainly in germinating seeds and growing seedlings of *A. thaliana* (Eastmond, 2006; Quettier and Eastmond, 2009; Kelly et al., 2011). LDs accumulation during seed development is critical for germination and seedling growth later on (Huang, 1996; Graham, 2008; Zienkiewicz A. et al., 2011; Zienkiewicz et al., 2014b). Based on the analysis of TAGs breakdown in Arabidopsis *sdp1*, *sdp1L*, and *sdp1 sdp1L* mutants it was demonstrated that among these two TAG lipases only *At*SDP1 (SUGAR DEPENDENT 1) plays a key role in mobilization of TAGs from LDs during seed germination (Eastmond, 2006; Kelly et al., 2011). In addition, the loss of *SDP1* function caused increased TAG content in the seeds of Arabidopsis (van Erp et al., 2014), rapeseed (*Brassica napus* L.) (Kelly et al., 2013a), Jatropha (*Jatropha curcas*) (Kim et al., 2014), and soybean (*Glycine max* L.) (Kanai et al., 2019), suggesting that SDP1 might be involved in TAGs turnover in developing seeds as well. Indeed, a decrease in oil content has often been observed during desiccation phase of seed development in some species (Baud et al., 2002; Chia et al., 2005; Kelly et al., 2013a). It has been proposed that at early stages of post-germinative growth, the inactive form of *At*SDP1 first localizes to the surface of peroxisomes and is then delivered to LDs *via* peroxules, initiating TAGs hydrolysis (**Figure 2**) (Thazar-Poulot et al., 2015; Cui et al., 2016; Esnay et al., 2020). The released FAs are then transported into peroxisomes by *At*PXA1 (an ATP-binding cassette (ABC) transporter) and enter the β-oxidation process (Zolman et al., 2001). Interestingly, Arabidopsis *sdp1* and *sdp1 sdp1L* mutants exhibit residual TAG hydrolysis on sugar-deficient medium, suggesting that additional lipases could also be involved in this process (Kelly et al., 2011). Therefore, the mobilization of TAGs was also analyzed in an Arabidopsis mutant with a lesion in *ADIPOSE TRIGLYCERIDE LIPASE-LIKE* (*ATGL*). *ATGL* encodes a lipase similar to human ATGL and COMPARATIVE GENE IDENTIFIER-58 (CGI-58), which possess TAG lipase, phospholipase A (PLA), and lysophosphatidic acid acyltransferase (LPAAT) activities (Eastmond, 2006; Ghosh et al., 2009; Kelly et al., 2011). However, no significant difference in the rate of TAGs breakdown was observed between wild type plants and these single mutants (Kelly et al., 2011). It was reported recently that *At*OBL1, a homolog of acid lipase from *Ricinus communis* (*Rc*OBL1, oil body lipase 1, Eastmond, 2004) possesses activity towards TAGs in Arabidopsis seeds (Müller and Ischebeck, 2018). Although *At*OBL1 is able to cleave TAGs and is associated with LDs, seed germination and TAGs breakdown rates in the *obl1* mutant were similar to those observed in wild type plants (Müller and Ischebeck, 2018). A specific LD lipoxygenase (LOX) and phospholipase A (PLA) seem to be involved in an alternative pathway of seed LDs degradation (Rudolph et al., 2011). LOX activity leads to formation of (9Z,11E,13S)-13-hydroperoxy octadeca-9,11-dienoic acid (13-HPOD) by oxygenation of linoleate moieties (18:2) of TAGs mobilized from LDs. The 13-HPOD liberated from TAGs can be later reduced to 13-HOD by the peroxygenase activity of caleosin (Rudolph et al., 2011). The LOX and PLA activities were detected *in vitro* on isolated LDs of cucumber (*Cucumis sativus*) (Rudolph et al., 2011) and olive (*Olea europaea* L.) (Zienkiewicz et al., 2014b) during seed germination. Thus, it was proposed that PLA might be responsible for LD membrane degradation and thereby facilitate access of LOX and lipase to TAG.

Pollen development, germination, and pollen tube growth are essential for sexual reproduction in flowering plants. During pollen hydration LDs polarize near the germinative aperture, and as the pollen grain starts to germinate, they enter the emerging pollen tube where their progressive degradation takes place (Zienkiewicz et al., 2013; Taurino et al., 2018). *At*SDP1L, unlike other putative TAG lipases identified in Arabidopsis, showed exceptionally high expression in mature pollen grains (Kelly et al., 2011). This suggests that *At*SDP1L may be involved in LDs breakdown during pollen germination. Two homologs of *Rc*OBL1, TAG lipase from tobacco (*Nt*OBL1) and *At*OBL1 from Arabidopsis mentioned above, have been characterized in pollen tubes (Müller and Ischebeck, 2018). Interestingly, similar to *Rc*OBL1 and unlike *At*SDP1 in seeds, both proteins localized to LDs when ectopically expressed in growing tobacco pollen tubes. Moreover, Arabidopsis *obl1* mutants showed slower pollen tube growth *in vivo*. The authors suggested that acyl groups released from TAGs by OBL1 might be directly channeled into the ER, where they serve as substrates for rapid membrane synthesis (Müller and Ischebeck, 2018).

LD-associated lipase activities were also found *in vitro* and *in situ* in germinating pollen and growing pollen tubes of olive (*Olea europaea* L.) (Rejon et al., 2012; Zienkiewicz et al., 2013). Their direct role in proper pollen tube growth has been confirmed by *in vitro* olive pollen germination in medium with and without sucrose, with higher lipase activity resulting in the latter case (Zienkiewicz et al., 2013). In these studies, TAG lipase activity was found only on LDs isolated from germinating pollen tubes, and not from the mature pollen grain. Similar localization pattern was also found for LOX protein (Zienkiewicz et al., 2013). In turn, PLA activity co-localized with LDs in both mature and germinating olive pollen (Zienkiewicz et al., 2013). This indicates that there are substantial differences in the temporal and spatial localization pattern of machinery governing LDs breakdown in pollen grains compared to seeds, where all the lipid degrading enzymes seem to be recruited to the LDs surface during or just after seed imbibition (Rudolph et al., 2011; Thazar-Poulot et al., 2015). Most probably, this reflects the distinct energy demands of pollen grains and seeds, which are tightly connected with their biological functions. The initiation of pollen germination and pollen tube growth occur much faster than seed imbibition and germination. This could explain the presence of some LDs degrading enzymes “on-site,” available to act as soon as pollen germination starts.

Previous studies have shown that disruption of *At*SDP1 also leads to TAG accumulation in other vegetative tissues, such us leaves, stems, and roots (Kelly et al., 2013b; Fan et al., 2014). Moreover, the expression level of *AtSDP1* increases during natural leaf senescence, thus it is possible that this lipase is involved in regulation of TAG homeostasis during leaf development (Troncoso-Ponce et al., 2013). The transcript levels corresponding to *AtCGI-58* were up-regulated during leaf senescence as well (Troncoso-Ponce et al., 2013), and loss of function of *At*CGI-58 resulted in a significant increase in TAG content of leaf mesophyll cells (James et al., 2010). While in mammals CGI-58 has been shown to act as an activator of adipose triglyceride lipase ATGL, and thus to directly regulate TAG breakdown (Lass et al., 2006), such a role has not been confirmed for plants, where CGI-58 likely regulates the activity of PXA1 but not of TAG lipase (Park et al., 2013). Consequently, the plant CGI-58 does not seem to be a bona fide TAG lipase, but rather an element of regulatory circuit of TAG homeostasis. Importantly, many putative lipases are expressed during leaf senescence (Troncoso-Ponce et al., 2013), suggesting more lipases could be involved in LD turnover in leaves.

LDs mobilization is an essential step during the transition of algal cells from quiescence to autotrophy in response to restored favorable growth conditions (Siaut et al., 2011; Tsai et al., 2018; Zienkiewicz et al., 2020). Thus, comparative transcriptomics between stress (N deprivation) and optimal (N resupply) growth conditions led to the identification of many putative TAG lipases highly expressed after triggering of the massive TAG degradation (Jaeger et al., 2017; Zienkiewicz et al., 2020). However, only a few of them have been functionally characterized to date (**Table 1**). Among 49 putative lipases encoded by the genome of the oleaginous marine diatom *P. tricornutum*, only one patatin-SDP1-like lipase, named tgl1, has been functionally characterized to date (Barka et al., 2016). The TAG lipase activity of tgl1 has been confirmed *in vitro* and *in vivo*. In the latter case, the *tgl1* knockdown mutants of *P. tricornutum* accumulated much higher amounts of TAGs when compared to the wild type (Barka et al., 2016). In *C. reinhardtii*, among over 130 putative lipases encoded by its genome, only one TAG lipase has been identified and characterized recently. This TAG lipase, LIP4, shares 44% amino acid identity with *At*SDP1 (**Table 1**) (Warakanont et al., 2019). The expression of *LIP4* is downregulated in response to N deprivation and up-regulated after N resupply, whereas its mutation resulted in delay of TAG degradation, which consequently led to TAG over-accumulation in *Crlip4* mutant (Warakanont et al., 2019). Two homologs of *At*SDP1, named *No*TGL1 and *No*TGL2, have also been recently reported in *N. oceanica* (Nobusawa et al., 2019). Over-accumulation of TAGs was observed only in the *N. oceanica* knockout of *NoTGL1*, but not of *NoTGL2*, after N resupply. Moreover, *No*TGL1 was found to be a specific resident of ER, thus the authors proposed its involvement in degradation of TAG *de novo* synthesized in the ER (Nobusawa et al., 2019). Proteomic analysis of another oleaginous green alga, *L. incisa*, revealed the presence of the putative TAG lipase *Li*SDP1. *Li*SDP1 shares 44% identity with *At*SDP1 (Siegler et al., 2017). Ectopic expression of *LiSDP1* in *A. thaliana sdp1 sdp1-L* mutants resulted in only partial complementation of the TAG lipase-deficient plants. *Li*SDP1 from *L. incisa* was absent in LD isolates from N-deprived cultures and did not localize to LDs when ectopically expressed in tobacco pollen tube. The authors did not exclude that *Li*SDP1 might be specifically recruited to the surface of LDs when needed (Siegler et al., 2017). By searching for human lipase homologs in the genome of *Thalassiosira pseudonana*, Trentacoste et al. (2013) identified a homolog of CGI-58, encoded by *Thaps3_264297*. The resulting protein has been shown to possess TAG lipase, PLA, and LPAAT activities *in vitro*, and a knockdown of *Thaps3_264297* resulted in increased TAG content and accumulation of LDs compared to the wild type strain (Trentacoste et al., 2013). However, taking into account the proposed role of plant CGI-58 the exact role of algal CGI-58 still needs to be elucidated.

**Table 1:** Functionally characterized TAG lipases involved in LDs degradation identified in plants and algae.

Organism	TAG Lipase	Reference
**Plants**		
*Arabidopsis thaliana* *Brassica napus* L. *Glycine max* L. *Jatropha curcas*	SDP1—Sugar Dependent 1	Eastmond, 2006; Kelly et al., 2011; Kelly et al., 2013a; Kim et al., 2014; Kanai et al., 2019
*Arabidopsis thaliana*	OBL1—Oil Body Lipase 1	Müller and Ischebeck, 2018
**Algae**		
*Chlamydomonas reinhardtii*	LIP4	Warakanont et al., 2019
*Lobosphaera incisa*	SDP1—Sugar Dependent 1	Siegler et al., 2017
*Nannochloropsis oceanica*	TGL1 and TGL2	Nobusawa et al., 2019
*Phaeodactylum tricornutum*	Tgl1	Barka et al., 2016

## Lipophagy—A New Player in The Field

Autophagy is a highly conserved process involved in regulation of intracellular degradation and recycling of individual molecules as well as whole organelles, *via* lysosomes in animals or vacuoles in yeast and plants (Avin-Wittenberg et al., 2012; Bento et al., 2016; Marshall and Vierstra, 2018; Couso et al., 2018). In plants, autophagy plays an important role in many developmental processes, such as seed development (Di Berardino et al., 2018; Sera et al., 2019) and leaf senescence (Avila-Ospina et al., 2014; Li et al., 2014). Autophagy is highly induced by various abiotic stresses, including nutrient deprivation (Sun et al., 2018; Janse van Rensburg et al., 2019), drought (Bao et al., 2020), and biotic stresses e.g. pathogen infection (Dagdas et al., 2016). Two major types of autophagy have been described in plants: macroautophagy and microautophagy (**Figure 2**). During macroautophagy, numerous autophagy-related (ATG) proteins participate in the induction of autophagy and formation of a double-membrane structure called an autophagosome (Yoshimoto and Ohsumi, 2018). Autophagosomes containing different cargo (macromolecules or damaged organelles) fuse with the tonoplast and subsequently enter the vacuolar lumen as autophagic bodies. The content of autophagic bodies then undergoes progressive degradation (Soto-Burgos et al., 2018). In microautophagy, cytoplasmic contents are directly captured into the vacuolar lumen by invagination of the tonoplast. Previous studies in yeast and mammalian cells demonstrated a close relationship between LD homeostasis and autophagy (van Zutphen et al., 2014; Petan et al., 2018). Indeed, autophagy may either provide FAs for LDs biogenesis (Rambold et al., 2015) or participate in their degradation in the process of lipophagy (Singh et al., 2009; van Zutphen et al., 2014). In animals, depending on the size of LDs, their degradation can be achieved either by macrolipophagy, where small, entire LDs are enclosed together with other cytoplasmic contents in an autophagosome, or by piecemeal microlipophagy, where only a small portion of a large LD is trapped in an autophagosome and then pinches off as a double membrane autolipophagosome (Singh et al., 2009; Khaldoun et al., 2014; Garcia et al., 2018). In yeast, distinct forms of microlipophagy contribute to LD degradation depending on growth conditions (van Zutphen et al., 2014; Vevea et al., 2015; Seo et al., 2017). For example, under acute glucose starvation, the molecular machinery of macroautophagy participates in the induction of microlipophagy. ATG14 is recruited to liquid-ordered membrane (Lo) domains at the surface of vacuole, where together with ATG6 it forms recruitment sites for LDs and initiates their microlipophagy (Seo et al., 2017). In contrast, under lipid and ER stress, microlipophagy depends on ESCRT (endosomal sorting complexes required for transport) machinery rather than ATG proteins (Vevea et al., 2015). Similarly, microlipophagy induced after diauxic shifts has been shown to be independent of the core ATGs but dependent on ESCRT proteins (Oku et al., 2017). Our knowledge on LDs TAG degradation inside the vacuole/lysosome is rather scarce and fragmentary. Nevertheless, one of the few reports showed that vacuolar lipase ATG15 acts in degradation of neutral lipids of LDs after their incorporation into the vacuole in yeast (Maeda et al., 2015).

Over the last decade research has also produced increasing evidence of an important role for autophagy in LDs degradation in plants (Kurusu et al., 2014; Fan et al., 2019) and algae (Zhao et al., 2014; Schwarz et al., 2017; Tsai et al., 2018). In plants, lipophagy seems to be involved among others in male reproductive development, pollen germination, and pollen tube growth (Kurusu et al., 2014; Hanamata et al., 2019; Hanamata et al., 2020; Zhao et al., 2020). The potential role of lipophagy in pollen grain maturation was demonstrated in a study on the *Oryza sativa* *atg7* mutant (Kurusu et al., 2014). Mutation in *OsATG7* was associated with a lower number of LDs in mature pollen and a higher accumulation of LDs in tapetal cells compared to wild type plants. These results, together with the observation that in wild type plants LDs were enclosed in the vacuoles of rice tapetal cells, suggest that ATG7-dependent tapetal autophagy may be responsible for LDs degradation and lipid metabolism in the tapetum (Kurusu et al., 2014). Recently, a detailed analysis of the germinating pollen lipidome in tobacco was performed using ATG-suppressed RNAi lines (Zhao et al., 2020). This study showed that silencing of *ATG2* and *ATG5* leads to a decrease in the number of autophagosomes at the germinative pollen aperture and is accompanied by inhibition of pollen germination and significant accumulation of TAGs and DAGs in pollen grains. Lipophagy may also contribute to the degradation of LDs during seed germination and seedling growth. The potential implication of autophagy in LDs degradation during seed germination was suggested by earlier studies of the Arabidopsis *clo1* mutant (Poxleitner et al., 2006). During seed germination, LDs were commonly observed in the vacuolar lumen of wild type cotyledon cells, while loss of function of *AtCLO1* resulted in the absence of LDs inside the vacuoles and slower degradation of eicosenoic acid (20:1) (Poxleitner et al., 2006). It is therefore possible that turnover of some pools of LDs is regulated by caleosin-dependent microlipophagy during seed germination. Other data showed that during *in vitro* germination of olive seeds, the neutral lipids stained by Sudan Black B co-localize in the area of protein bodies (known also as protein storage vacuoles), together with lipase activity, suggesting that LDs degradation takes place inside protein bodies (Zienkiewicz et al., 2014b). A significant accumulation of TAGs was observed in etiolated carbon-starved seedlings of the Arabidopsis *atg5* mutant (Avin-Wittenberg et al., 2015), indicating the potential role of autophagic machinery in LDs degradation. It was demonstrated recently that LDs breakdown in senescent watermelon (*Cirullus lanatus*) leaves occurs *via* different pathways: small vacuole-associated and central vacuole-associated (Zhang et al., 2020). In leaf cells without a central vacuole, LDs interact with autophagosome-like structures before they enter small vacuoles. Meanwhile, in the cells of senescent leaves containing the central vacuole, LDs are delivered into the vacuolar lumen *via* a process morphologically resembling microlipophagy (Zhang et al., 2020). Lipophagy has also been implicated in LD turnover in Arabidopsis leaves during dark-induced starvation (Fan et al., 2019). Under extended darkness, DsRed-Atg8e-labeled autophagic structures were observed to be associated with LDs in the leaves of *tgd1* (*trigalactosyldiacylglycerol1*) mutant. Moreover, ultrastructural analysis of leaf cells from dark-treated *Atsdp1-4* plants, deficient in cytosolic lipolysis, demonstrated the entrance and appearance of LDs in the central vacuole. The authors suggested that this process is likely mediated by the microlipophagy pathway (Fan et al., 2019). Interestingly, disruption of molecular macroautophagy machinery in Arabidopsis double mutants *atg2-1 sdp1-4* and *atg5-1 sdp1-4* leads to inhibition of dark-induced lipophagy in the leaves. Taken together, the findings described by Fan et al. (2019) showed that microlipophagy observed in Arabidopsis leaves depends on core components of macroautophagy pathway, as has been described previously in yeast (van Zutphen et al., 2014).

A large number of studies on autophagy-mediated LDs degradation in algae have been performed using *C. reinhardtii* as a reference. Ultrastructural analysis of *C. reinhardtii* cells under N resupply (NR) conditions showed the appearance of small LDs in the vacuolar lumen is controlled by a process morphologically resembling microlipophagy (Tsai et al., 2018). A mutation in *CrATG8*, encoding one of the core ATG proteins required for the formation of the autophagosome, results in delayed degradation of TAGs after NR compared to wild type lines (Kajikawa et al., 2019). In addition, by using mCherry-ATG8 as a tool to monitor the intracellular movements of autophagosomes (Yoshimoto et al., 2004; Klionsky et al., 2007), the fusion between autophagosomes and LDs was observed at later stages of N deprivation (Tran et al., 2019). Similar to *C. reinhardtii*, small LDs were observed to be sequestered by the vacuole *via* a pathway resembling microautophagy in another green alga, *Auxenochlorella protothecoides*, during its heterotrophy to autotrophy transition (Zhao et al., 2014). Interestingly, the authors suggested that small portions of large LDs might also be degraded in the vacuolar lumen through a process reminiscent of the piecemeal microautophagy commonly observed in mammalian cells. Microlipophagy-like degradation of LDs was observed as well in *N. oceanica* under NR conditions (Zienkiewicz et al., 2020). Moreover, by using a biomolecular fluorescence complementation (BiFC) assay it was demonstrated that *No*ATG8 protein interacts *in vivo* with *No*LDSP —a major LD surface protein in *N. oceanica* (Zienkiewicz et al., 2020). It has been proposed that this interaction might be involved in the targeting and/or fusion of LDs into the vacuole. The ATG8-interacting motif (AIM) was also identified in Stramenopile-type lipid droplet protein (StLDP), suggesting a possible link between autophagy and LD degradation in *P. tricornutum* (Leyland et al., 2020). The latest analyses revealed that salt stress is also associated with appearance of LDs in the vacuolar lumen in *Parachlorella kessleri* (You et al., 2019). Interestingly, in the unicellular alga *Micrasterias denticulate*, LDs degradation occurring under carbon starvation seems to be mediated by macrolipophagy, as LDs were trapped in autophagosomes and then delivered into small vacuoles (Schwarz et al., 2017).

## LD Protein Turnover—The Missing Puzzle In The Crosstalk Between Lipolysis And Lipophagy?

Previous studies in animals revealed that association of ATGL with the surface of LDs depends on the degradation of the LDs structural proteins perilipin 2 (PLIN2) and perilipin 3 (PLIN3) *via* chaperone-mediated autophagy (CMA) (Kaushik and Cuervo, 2015). Sathyanarayan et al. (2017) proposed a model where the removal of PLINs facilitates access of ATGL to TAGs, and ATGL acts as a signal for LDs recognition by autophagosomes and thus an inducer of bulk LDs degradation *via* lipophagy (Sathyanarayan et al., 2017). In plants, oleosins and LDAPs have been proposed to protect LDs from the action of TAG lipases (Siloto et al., 2006; Gidda et al., 2016). Recently, two independent groups reported that ubiquitinated oleosins interact with the PUX10 protein through its UBA domain. *Via* its UBX domain, PUX10 in turn recruits CDC48, enabling further degradation of oleosins by the proteasome (Deruyffelaere et al., 2018; Kretzschmar et al., 2018). On the other hand, it has been found that *At*OLE1 can interact with *At*ATG8e protein through the AIM motif (ATG8 interacting motif) (Marshall et al., 2019). This implies that autophagy can also participate in oleosin turnover during seed germination. Interestingly, ATGL and hormone-sensitive lipase (HSL) contain several putative LIR (LC3-interacting region) motifs responsible for direct interaction with microtubule associated protein 1 LC3 (ATG8 family protein) (Martinez-Lopez et al., 2016). Mutating the ATGL LIR motif blocked ATGL associations with the LD surface and lipolysis, suggesting the direct crosstalk between lipolysis and autophagy in animal cells. It remains unknown if the degradation of plant and algal TAG lipases is also controlled by autophagy machinery.

## Concluding Remarks

The past decade has seen considerable progress in the identification and characterization of mechanisms governing LDs degradation in plant and algal cells. The studies reviewed here revealed the complexity of intracellular molecular networks related to LDs metabolism, and highlighted the fundamental meaning of these organelles for developmental programs and physiological responses in plants and algae. This includes essential functions of LDs structural proteins (oleosins, celeosins, and their counterparts in algal cells) as well as lipolysis and lipophagy pathways of TAGs breakdown. However, there are still many unanswered questions. For example, what is the nature of cross-talk between lipolysis and lipophagy in plant and algal cells? And what specific lipophagy receptors on the surface of LDs could be involved in activation of the autophagic machinery and lipophagy initiation? Moreover, it is still unclear whether, similar to animal cells, plant and algal TAG lipases are implicated in the induction of lipophagy, and if autophagy is responsible for their degradation. Answering these questions will help us gain a better comprehensive understanding of induction and regulation of LDs mobilization in plants and algae. Future research is needed to uncover the fascinating and multifaceted mechanisms that govern LDs turnover in plant and algal cells, as this knowledge is crucial for the development of more applied research and engineering of lipid-rich biomass production from algae and oil crops.

## Author Contributions

KZ and AZ conceived, wrote the manuscript and designed the figures. All authors contributed to the article and approved the submitted version.

## Conflict of Interest

The authors declare that the research was conducted in the absence of any commercial or financial relationships that could be construed as a potential conflict of interest.

## Supplementary Material

Supplementary Table 1 LD-associated proteins identified in plants and algae. The corresponding references are given next to the table.
